# Antioxidant activities and phenolic contents of the methanol extracts of the stems of *Acokanthera oppositifolia *and *Adenia gummifera*

**DOI:** 10.1186/1472-6882-8-54

**Published:** 2008-09-25

**Authors:** Adeolu A Adedapo, Florence O Jimoh, Anthony J Afolayan, Patrick J Masika

**Affiliations:** 1 Department of Veterinary Physiology, Biochemistry and Pharmacology, University of Ibadan, Ibadan, Nigeria; 2 Department of Botany, University of Fort Hare, Alice 5700, South Africa; 3 ARDRI, University of Fort Hare, Alice 5700, South Africa

## Abstract

**Background:**

*Acokanthera oppositifolia *Lam (family: Apocynaceae) is a shrub or small tree with white latex, and the leaves of this plant are used in the form of a snuff to treat headaches and in infusions for abdominal pains and convulsions and septicaemia. *Adenia gummifera *Harv of the family Passifloraceae is a distinctive woody climber whose infusions are used as emetics and are said to help with some forms of depression. Lipid peroxidation has gained more importance today because of its involvement in pathogenesis of many diseases. Free radicals are the main agents in lipid peroxidation. Antioxidants thus play an important role of protecting the human body against damage by the free radicals. Plants containing phenolic compounds have been reported to possess strong antioxidant properties.

**Methods:**

The antioxidant activities and phenolic contents of the methanol extracts of the stems of *Acokanthera oppositifolia *and *Adenia gummifera *were evaluated using *in vitro *standard procedures. Spectrophotometry was the basis for the determinations of total phenol, total flavonoids, flavonols, and proanthocyanidins. Tannins, quercetin and catechin equivalents were used for these parameters. The antioxidant activities of the stem extract of *Acokanthera oppositifolia *were determined by the 2,2'-azinobis-3- ethylbenzothiazoline-6-sulfonic acid (ABTS), 1,1-Diphenyl-2-picrylhydrazyl (DPPH), and ferrous reducing antioxidant property (FRAP) methods.

**Results:**

The results from this study showed that the antioxidant activities of the stem extract of *Acokanthera oppositifolia *as determined by the 1,1-Diphenyl-2-picrylhydrazyl (DPPH), and ferrous reducing antioxidant property (FRAP) methods, were higher than that of *Adenia gummifera*. The levels of total phenols and flavonols for *A. oppositifolia *were also higher. On the other hand, the stem extract of *Adenia gummifera *had higher level of total flavonoids and proanthocyanidins than that of *Acokanthera oppositifolia*. The 2, 2'-azinobis-3- ethylbenzothiazoline-6-sulfonic acid (ABTS) activities of the 2 plant extracts were similar and comparable to that of BHT.

**Conclusion:**

Thus, the present results indicate clearly that the extracts of *Acokanthera oppositifolia *and *Adenia gummifera *possess antioxidant properties and could serve as free radical inhibitors or scavengers, acting possibly as primary antioxidants. This study has to some extent validated the medicinal potential of the stems of *Acokanthera oppositifolia *and *Adenia gummifera*.

## Background

*Acokanthera oppositifolia *Lam (family: Apocynaceae) is a shrub or small tree with white latex, thick leathery leaves, attractive white flowers and red berries which turn dark purple when ripen. The latex, fruit and decoctions of the wood of this plant were widely used as arrow poisoning in southern Africa. These plant parts can sometimes be combined with *Euphorbia *latex, the sap of *Acacia mellifera *and the venom from the poison glands of snake and used as arrow poisoning. In the Northern Cape of South Africa, arrows poisoned with Acokanthera and snake venom were used to kill antelope and buffalo, and against enemies [1-4]. Poisoning of animals by this plant is surprisingly rare but cattle are sometimes at risk during droughts [5].

The leaves of this plant are used in the form of a snuff to treat headaches and in infusions for abdominal pains and convulsions and septicaemia. Powdered roots are administered orally or as snuff to treat pain and snake-bite and root decoctions are used against anthrax and tapeworm [4,6,7]. The leaves of this plant when boiled in water for ten minutes, strained and left to stand overnight are given to goats and sheep (200 ml) to treat heart water disease [7]. Members of the genus Acokanthera contain several toxic cardiac glycosides such as ouabain [4,8,9]. Acovenoside, a cardiac glycoside, is the major toxic component of both *A. oppositifolia *and *A. oblongifolia *[4].

*Adenia gummifera *Harv of the family Passifloraceae is a distinctive woody climber with bright green stems and lobed leaves. Infusions are used as emetics and are said to help with some forms of depression. Though the thick, green stem is said to be very poisonous but is popular for treating of leprosy and malaria [4,6]. Species of *Adenia *have been used as fish poisons [2] and have also been implicated in stock losses, homicide and suicide [1,2,4,5]. The toxicity of Adenia species is due to the combination of a highly toxic protein, modeccin, and cyanogenic glycosides [4,10-12]. Gummiferol, a cytotoxic polyacetylenic diepoxide was isolated from the leaves of *Adenia gummifera *by KB cytotoxicity-guided fractionation and this compound exhibited significant activity against the KB human cell line and a broad cytotoxic spectrum against other human cancer cell lines [13]. KB or NFKB is nuclear activated kappa B, and is a transcription factor that has a key role in the induction of inflammatory and immune response [14].

Lipid peroxidation has gained more importance today because of its involvement in pathogenesis of many diseases like atherosclerosis, cancer, diabetes mellitus, myocardial infarction, and also ageing. Free radicals or reactive oxygen species (ROS) are produced *in vivo *from various biochemical reactions and also from the respiratory chain as a result of occasional leakage. These free radicals are the main agents in lipid peroxidation [15]. Antioxidants thus play an important role of protecting the human body against damage by reactive oxygen species [16,17]. Plants containing phenolic compounds, in particular flavonoids have been reported to possess strong antioxidant properties [18,19].

In the present study, the methanol extracts of the stem of *Acokanthera oppositifolia *and *Adenia gummifera *were screened for antioxidant properties using *in vitro *standard procedures so as to assess the medicinal potential of these 2 plants and thus justify their folklore use.

## Methods

### Plant collection

The plants were collected in July 2006 from some villages in the Eastern Cape Province of South Africa. The area falls within the latitudes 30°00–34° 15'S and longitudes 22° 45' -30° 15'E. It is bounded by the sea in the east and the drier Karoo (semi-desert vegetation) in the west [20]. These areas consist of villages which are generally classified as rural and poor. The plants were identified by their vernacular names and later validated at the Department of Botany, University of Fort Hare and voucher specimens (Aded Med 2007/1-10) were deposited in the Griffen Herbarium of the University.

### Extract preparation

Plants were air dried at room temperature for 3 weeks to get consistent weight. The dried plants were later ground to powder. Two hundred grams of ground plant material were shaken separately in methanol for 48 hrs on an orbital shaker at room temperature. Extracts were filtered using a Buckner funnel and Whatman No 1 filter paper. Each filtrate was concentrated to dryness under reduced pressure at 40°C using a rotary evaporator. Each extract was resuspended in the respective solvent, methanol, to yield a 50 mg/ml stock solution [21].

### Chemicals

1,1-Diphenyl-2-picrylhydrazyl (DPPH), 2,2'-azinobis-3- ethylbenzothiazoline-6-sulfonic acid (ABTS), 3-(2-pyridyl)-5,6-diphenyl-1,2,4-triazine-4',4"-disulfonic acid, potassium ferricyanide; catechin, butylated hydroxytoluene (BHT), ascorbic acid, catechin, tannic acid, quercetin and FeCl_3 _were purchased from Sigma Chemical Co. (St. Louis, MO, USA)., vanillin from BDH; Folin-Ciocalteus's phenol reagent and sodium carbonate were from Merck Chemical Supplies (Damstadt, Germany). All the other chemicals used including the solvents, were of analytical grade.

### Determination of total phenolics

Total phenolic contents in the extracts were determined by the modified Folin-Ciocalteu method [22]. An aliquot of the extracts was mixed with 5 ml Folin-Ciocalteu reagent (previously diluted with water 1:10 v/v) and 4 ml (75 g/l) of sodium carbonate. The tubes were vortexed for 15 sec and allowed to stand for 30 min at 40°C for color development. Absorbance was then measured at 765 nm using the Hewlett Packard UV-VIS spectrophotometer. Samples of extract were evaluated at a final concentration of 0.1 mg/ml. Total phenolic content was expressed as mg/g tannic acid equivalent using the following equation based on the calibration curve: *y *= 0.1216x, R^2 ^= 0.9365, where x was the absorbance and y was the tannic acid equivalent (mg/g).

### Determination of total Flavonoids

Total flavonoid contents were determined using the method of Ordon ez et al., [23] of sample solution. A volume of 0.5 ml of 2% AlCl_3 _ethanol solution was added to 0.5 ml of sample solution. After one hour at room temperature, the absorbance was measured at 420 nm. A yellow color indicated the presence of flavonoids. Extract samples were evaluated at a final concentration of 0.1 mg/ml. Total flavonoid content were calculated as quercetin (mg/g) using the following equation based on the calibration curve: *y *= 0.0255x, R^2 ^= 0.9812, where x was the absorbance and was the quercetin equivalent (mg/g).

### Determination of total Flavonols

Total flavonols in the plant extracts were estimated using the method of Kumaran and Karunakaran [24]. To 2.0 mL of sample (standard), 2.0 mL of 2% AlCl_3 _ethanol and 3.0 mL (50 g/L) sodium acetate solutions were added. The absorption at 440 nm was read after 2.5 h at 20°C. Extract samples were evaluated at a final concentration of 0.1 mg/ml. Total flavonoid content was calculated as quercetin (mg/g) using the following equation based on the calibration curve: y = 0.0255x, R^2 ^= 0.9812, where x was the absorbance and was the quercetin equivalent (mg/g).

### Determination of total proanthocyanidins

Determination of proanthocyanidin was based on the procedure reported by Sun et al., [25]. A volume of 0.5 ml of 0.1 mg/ml of extract solution was mixed with 3 ml of 4% vanillin-methanol solution and 1.5 ml hydrochloric acid; the mixture was allowed to stand for 15 min. The absorbance was measured at 500 nm. Extract samples were evaluated at a final concentration of 0.1 mg/ml. Total proanthocyanidin content were expressed as catechin equivalents (mg/g) using the following equation based on the calibration curve: y = 0.5825x, R^2 ^= 0.9277, where x was the absorbance and y is the catechin equivalent (mg/g).

### Determination of antioxidant activity

#### ABTS radical scavenging assay

For ABTS assay, the method of Re et al., [26] was adopted. The stock solutions included 7 mM ABTS solution and 2.4 mM potassium persulfate solution. The working solution was then prepared by mixing the two stock solutions in equal quantities and allowing them to react for 12 h at room temperature in the dark. The solution was then diluted by mixing 1 ml ABTS^.+ ^solution with 60 ml methanol to obtain an absorbance of 0.706 ± 0.001 units at 734 nm using the spectrophotometer. ABTS^.+ ^solution was freshly prepared for each assay. Plant extracts (1 ml) were allowed to react with 1 ml of the ABTS^.+ ^solution and the absorbance was taken at 734 nm after 7 min using the spectrophotometer. The ABTS^.+ ^scavenging capacity of the extract was compared with that of BHT and percentage inhibition calculated as ABTS radical scavenging activity (%) = [(Abs_control _– Abs_sample_)]/(Abs_control)_] × 100 where Abs_control _is the absorbance of ABTS radical + methanol; Abs_sample _is the absorbance of ABTS radical + sample extract/standard.

#### DPPH radical scavenging assay

The effect of extracts on DPPH radical was determined using the method of Liyana-Pathiranan & Shahidi [27]. A solution of 0.135 mM DPPH in methanol was prepared and 1.0 ml of this solution was mixed with 1.0 ml of extract in methanol containing 0.02–0.1 mg of the extract. The reaction mixture was vortexed thoroughly and left in the dark at room temperature for 30 min. The absorbance of the mixture was measured spectrophotometrically at 517 nm. Ascorbic acid and BHT were used as references. The ability to scavenge DPPH radical was calculated by the following equation: DPPH radical scavenging activity (%) = [(Abs_control _– Abs_sample_)]/(Abs_control)_] × 100 where Abs_control _is the absorbance of DPPH radical + methanol; Abs_sample _is the absorbance of DPPH radical + sample extract/standard.

#### Total antioxidant activity (FRAP assay)

A modified method of Benzie & Strain [28] was adopted for the FRAP assay. The stock solutions included 300 mM acetate buffer (3.1 g CH_3_COONa and 16 ml CH_3_OOH), pH 3.6, 10 mM TPTZ (2, 4, 6-tripyridyl-*s*-triazine) solution in 40 mM HCl, and 20 mM FeCl_3_·6H_2_O solution. The fresh working solution was prepared by mixing 25 ml acetate buffer, 2.5 ml TPTZ, and 2.5 ml FeCl_3_·6H_2_O. The temperature of the solution was raised to 37 °C before using. Plant extracts (150 μL) were allowed to react with 2850 μl of the FRAP solution for 30 min in the dark condition. Readings of the colored product (ferrous tripyridyltriazine complex) were taken at 593 nm. The standard curve was linear between 200 and 1000 μM FeSO_4_. Results are expressed in μM Fe (II)/g dry mass and compared with that of BHT, ascorbic acid and catechin.

### Statistical analysis

The experimental results were expressed as mean ± standard error of mean (SEM) of three replicates. Where applicable, the data were subjected to one way analysis of variance (ANOVA) and differences between samples were determined by Duncan's Multiple Range test using the Statistical Analysis System (SAS 1999) program. *P *Values < 0.05 were regarded as significant and *P *values < 0.01 as very significant.

## Results

### Total phenolic, flavonoids, flavonols, and proanthocyanidin contents

Results obtained in the present study revealed that the level of these phenolic compounds in *A. gummifera *and *A. oppositifolia *was significant with the extract from the stem of A. oppositifolia showing higher level of phenolic compounds (Table 1). When compared to the standard compounds used in this study, the levels in the plant extracts are significantly lower.

**Table 1:** Polyphenol contents of the methanol extracts of the stems of *A. oppositifolia *and *Adenia gummifera*.

Phenolics	*A. oppositifolia*	*A. gummifera*
Total polyphenol^a^	9.51 ± 2.12	8.24 ± 0.77
Flavonoids^b^	0.81 ± 0.02	1.11 ± 0.02
Total Flavonol^c^	1.01 ± 0.37	1.11 ± 0.02
Proanthocyanidins^d^	0.71 ± 0.20	1.14 ± 0.31

(n = 3, X ± SEM).

^a^Expressed as mg tannic acid/g of dry plant material.

^b^Expressed as mg quercetin/g of dry plant material.

^c^Expressed as mg quercetin/g of dry plant material.

^d^Expressed as mg quercetin/g of dry plant material

### Total antioxidant power (FRAP)

The reducing ability of the extracts was in the range of 159.12 – 301.21 mm Fe (II)/g (Table 2). The FRAP values for the *A. gummifera *and *A. oppositifolia *extracts were significantly lower than that of ascorbic acid and catechin, but higher than that of BHT.

**Table 2:** Ferric reducing antioxidant property (FRAP) of the stem extracts of *A. oblongifolia *and *A. gummifera*.

Extracts	FRAP (μmol Fe(II)/g)
*A. oblongifolia*	301.21 ± 12.96
*A. gummifera*	159.12 ± 7.58
Ascorbic acid	1632.1 ± 16.95
BHT	63.46 ± 2.49
Catechin	972.02 ± 0.61
Quercetin	3107.29 ± 31.28

(n = 3, X ± SEM).

### ABTS radical scavenging activity

**Figure 1:**
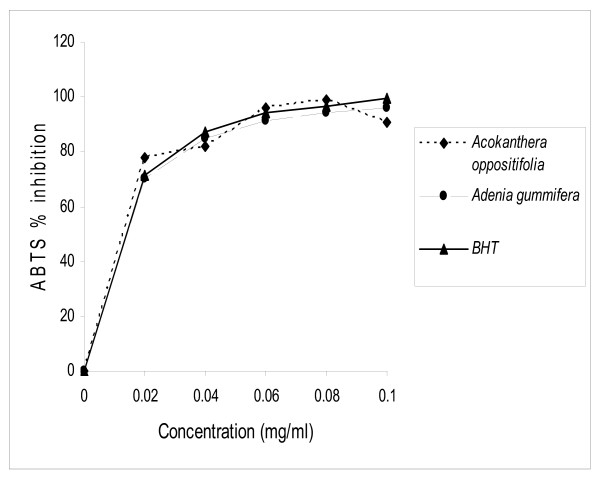
ABTS scavenging activity of the methanol extracts of the stems of *Acokanthera oppositifolia *and *Adenia gummifera*.

*A. oppositifolia *and *A. gummifera *extracts were fast and effective scavengers of the ABTS radical (Fig 1) and this activity was comparable to that of BHT. At 0.08 mg/ml the percentage inhibition was 99.0, 94.2 and 96.8% for *A. oppositifolia, A. gummifera *and BHT respectively. On the other hand, at 0.1 mg/ml, the percentage inhibition was 90.5, 95.5 and 99.3% for *A. oppositifolia, A. gummifera *and BHT respectively.

### DPPH radical scavenging activity

**Figure 2:**
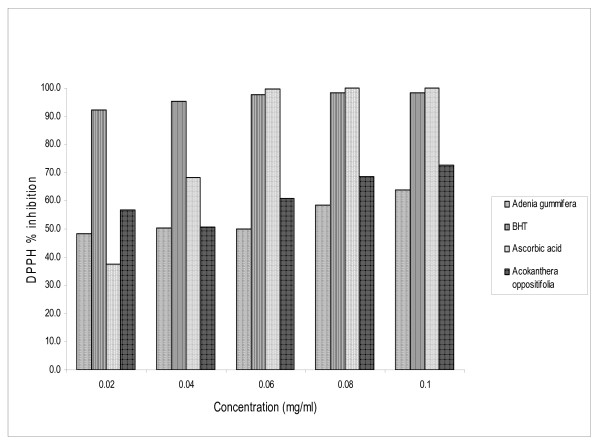
DPPH scavenging activities of *Acokanthera oppositifolia *and *Adenia gummifera*.

Figure 2 shows the dose-response curve of DPPH radical scavenging activity of the methanolic extracts of the stem of *Acokanthera oppositifolia *and *Adenia gummifera*, compared with BHT and ascorbic acid. It was observed that methanol extract of *A. oppositifolia *had higher activity than that of *A. gummifera*. At a concentration of 0.1 mg/ml, the scavenging activity of methanol extract of *A. oppositifolia *reached 70%, while that of *A. gummifera *methanol extract was only 60%.

## Discussion

### Total phenolic, flavonoids and proanthocyanidin contents

Polyphenols are the major plant compounds with antioxidant activity. This activity is believed to be mainly due to their redox properties [29], which play an important role in adsorbing and neutralizing free radicals, quenching singlet and triplet oxygen, or decomposing peroxides. The results from this study strongly suggest that phenolics are important components of these plants, and some of their pharmacological effects could be attributed to the presence of these valuable constituents.

### Total antioxidant power (FRAP)

The antioxidant potentials of *A. gummifera *and *A. oppositifolia *extracts were estimated from their ability to reduce TPRZ-Fe (III) complex to TPTZ-Fe (II). Antioxidant activity increased proportionally to the polyphenol content. According to recent reports, a highly positive relationship between total phenols and antioxidant activity appears to be the trend in many plant species [30].

### ABTS radical scavenging activity

Proton radical scavenging is an important attribute of antioxidants. ABTS, a protonated radical, has characteristic absorbance maxima at 734 nm which decreases with the scavenging of the proton radicals [31]. The 2,2'-azinobis-3- ethylbenzothiazoline-6-sulfonic acid (ABTS) activities of the 2 plant extracts were similar and comparable to that of BHT. Higher concentrations of the extracts were more effective in quenching free radicals in the system.

### DPPH radical scavenging activity

The effect of antioxidants on DPPH is thought to be due to their hydrogen donating ability [32]. Although the DPPH radical scavenging abilities of the extracts were significantly lower than those of ascorbic acid and BHT, it was evident that the extracts did show the proton-donating ability and could serve as free radical inhibitors or scavengers, acting possibly as primary antioxidants.

The scavenging of the ABTS^+ ^radical by the extracts was found to be higher than that of DPPH radical. Factors like stereoselectivity of the radicals or the solubility of the extract in different testing systems have been reported to affect the capacity of extracts to react and quench different radicals [33]. Wang et al., [34] found that some compounds which have ABTS^+ ^scavenging activity did not show DPPH scavenging activity. In this study, this was not the case. This further showed the capability of the extracts to scavenge different free radicals in different systems, indicating that they may be useful therapeutic agents for treating radical-related pathological damage.

## Conclusion

Although in most cases, the biological activities of the extracts from the stems of *A. oppositifolia *and *A. gummifera *are not as high as those of the standard compounds used in this study, the present results indicate clearly that the extracts from these plants possess antioxidant properties and could serve as free radical inhibitors or scavengers, acting possibly as primary antioxidants. This study has to some extent validated the medicinal potential of the stems of *Acokanthera oppositifolia *and *Adenia gummifera*.

## Competing interests

The authors declare that they have no competing interests.

## Authors' contributions

AAA: Prepare the extract, carried out the assays and drafted the manuscript. FOJ: Carried out the assay. AJA: Coordinated the study. PJM: Provided the grants for the study and also coordinated the study. All authors read and approved the final manuscript.

## Pre-publication history

The pre-publication history for this paper can be accessed here:


